# Artificial Intelligence-Based Predictive Modeling for Aortic Aneurysms

**DOI:** 10.7759/cureus.79662

**Published:** 2025-02-25

**Authors:** Ghulam Husain Abbas, Edmon Khouri, Sjaak Pouwels

**Affiliations:** 1 Faculty of Medicine, Ala-Too International University, Bishkek, KGZ; 2 Department of Medicine, Mass General Brigham, Boston, USA; 3 Faculty of Medicine, University of Jordan, Amman, JOR; 4 Department of Surgery, Bielefeld University - Detmold Campus, Detmold, DEU; 5 Department of Intensive Care Medicine, Elisabeth-Tweesteden Hospital, Tilburg, NLD

**Keywords:** abdominal aortic aneurysms, ai and machine learning, ai in cardiology, aortic aneurysm, aortic diseases, artificial intelligence, cardiology, cardiovascular medicine, machine learning, predictive modeling

## Abstract

Abdominal aortic aneurysms (AAAs) remain a major concern to the global society because of the associated risk of rupture and death. Currently, the management of AAAs entails clinical and imaging risk factors, which are not precise and accurate in terms of patient-specific risk assessment. Over the last decade, the utilization of artificial intelligence (AI) and machine learning (ML) algorithms has transformed the process of decision-making in the field of medicine by allowing for the creation of personalized models based on the patient’s characteristics. This review aims to discuss the current state and future directions of AI in the form of predictive modeling for aortic aneurysms, stressing the versatility and progression of the ML approaches in risk assessment, screening, and prognosis. We expand on the various strategies used in AI-based solutions and the differences between general and specific approaches such as supervised and unsupervised learning, deep learning, and others. Furthermore, we bring forward the problem of incorporating clinical, imaging, and genomic data into AI/ML to improve its predictiveness and applicability to clinical practice. In addition, we discuss the difficulties and prospects of turning the developed AI-based forecasting models into clinical practice, as well as the problems associated with data quality, model explainability, and legal and ethical concerns. This review aims to reveal the opportunities of AI and ML in enhancing the risk assessment and management of AAAs to shift the paradigm of cardiovascular care toward precision medicine.

## Introduction and background

With the advent of artificial intelligence (AI) and machine learning (ML) in the field of contemporary cardiology and vascular surgery, a new, complex approach is already opening, which will significantly change the methods of making decisions in clinical practice [1]. Thus, as there is a constantly growing pool of complex accumulated medical information, AI appears as a powerful tool that can differentiate between fine details and find patterns that other methods could miss. As for cardiovascular diseases, AI is most influential in the field because it alters the approaches to diagnostics, treatment, and risk assessment, particularly in cases of aortic aneurysms, in which timely diagnosis and intervention are critical [1]. Through deep learning and neural networks, cardiology and vascular surgery see a revival of diagnostic imaging where AI analyses provide more accurate and efficient diagnosis of cardio-related ailments [2].

Furthermore, with the increase in life expectancy of the world population and changes in lifestyles, the incidence of cardiovascular diseases is on the rise, and hence, there is a need to advance the healthcare industry [2]. AI and ML are emerging technologies that are contributing to advancements in healthcare delivery, particularly in vascular surgery. The following review attempts to embark on a voyage through the world of artificial intelligence and aortic aneurysms, exploring the multiplicity of possibilities and issues that these emerging technologies hold and create as they define the future of precision medicine and fuel novel clinical investigations [2].

To demystify the intricate processes that AI contributes to the advancement of cardiovascular care, this review aims to give a balanced depiction of the mutually reinforcing relationship between technology and medicine, envisioning the future in which artificial intelligence shapes the future of vascular surgery, providing new opportunities for improving patient conditions and raising the bar of medical practice [3].

The use of AI in vascular surgery is a major innovative solution; AI is already being used for early risk assessment and for diagnosing complicated cases of heart diseases. AI is involved in cardiology in several aspects, some of which are as follows [3]. First, AI systems, especially neural networks, are highly efficient when it comes to working with large sets of data to find subtle patterns, which plays a significant role in test interpretation and identification of different types of heart disorders, as well as in predicting potential cardiovascular problems in the future [3]. Moreover, in the field of cardiovascular medicine, the application of AI has shortened the time and expenses required for the prognosis of pharmacological activities of new molecules and is a major advancement in drug discovery and patient treatment [4].

In addition, AI-assisted image analysis algorithms provide fast and precise identification of distortions in medical images and, based on large patient records, predict the potential risk of cardiovascular diseases with greater accuracy. The improvement in forecasting heart-related incidents with the help of AI permits clinicians to manage applicable interventional strategies in detail, especially in higher-risk patients [3-4]. Furthermore, applications that integrate with AI perform well in terms of time and accuracy in medical images such as X-rays, MRI, and CT scans. These technologies help cardiologists localize an issue, comprehend the evolution and effects of diseases, and develop treatment procedures. For instance, research involved the use of a deep learning algorithm known as CheXNet to diagnose pneumonia from chest X-ray images [5]. This model revealed a very high level of accuracy and specificity in the identification of pneumonia compared to radiologists alone. While this capability is highly valued in vascular surgery for accurate imaging analysis, its direct relevance to abdominal aortic aneurysms (AAAs) remains limited unless adapted for aneurysm detection and monitoring [5].

While AI has shown great potential in the interpretation of ECGs and the early identification of arrhythmias, its direct application to AAA treatment is still evolving. AI's main contribution to vascular surgery, however, lies in improving imaging-based risk assessment and aneurysm growth and rupture prediction models. Accurate diagnosis and patient-specific risk assessment have been enhanced by machine learning models trained on extensive AAA datasets, which include morphometric and biomechanical features [6-7].

AI-driven medical diagnostics have transformed risk assessment, disease detection, and clinical decision-making. Technologies such as Aidoc's BriefCase enhance radiology workflows by prioritizing cervical spinal fractures, while Zebra Medical Vision’s HealthVCF improves vertebral compression fracture detection. AI has also optimized incidental pulmonary embolism detection, reducing diagnostic delays and treatment times [8]. In addition, DeepMind’s AI has demonstrated the ability to forecast acute kidney injury (AKI) days in advance [9]. While these advancements highlight AI's predictive capabilities, their direct application to AAA management is still under investigation. Future adaptations of such predictive technologies could further improve AAA risk stratification, growth assessment, and rupture forecasting.

Similarly, AI has improved fracture diagnosis, decreasing misdiagnoses and increasing detection accuracy [10]. These advancements in AI-driven diagnostics, risk prediction, and treatment guidance provide a framework for its potential application to AAAs. While AI in AAA management is still evolving, its success in radiology and nephrology suggests that it could be adapted for AAA risk prediction, growth assessment, and rupture forecasting. However, clinical validation and regulatory approval are essential steps before full-scale implementation in practice. Future research should explore AI’s role in real-time AAA monitoring and treatment planning, ensuring seamless clinical integration and physician acceptance.

In summary, the fusion of AI and ML with cardiology and vascular surgery is ushering in a new era of precision medicine. By leveraging these technologies, healthcare professionals can enhance diagnostic accuracy, personalize treatment plans, and improve patient outcomes in cardiovascular care. Although AI has already demonstrated transformative potential in various aspects of cardiovascular medicine, its application in AAA management remains an area of active research, requiring further advancements and validation to achieve widespread clinical adoption.

## Review

Overview of AI methods for predicting aortic aneurysms

Vascular surgery now heavily relies on AI, especially when it comes to predicting the growth and rupture risk of AAAs. Various AI methods, like convolutional neural networks (CNNs), deep learning, and XGBoost, have shown notable improvements in risk assessment, treatment planning, and diagnosis accuracy for AAA patients [11-12]. Deep learning improved diagnostic accuracy, this branch of ML uses artificial neural networks to evaluate intricate medical data. Deep learning algorithms have been used in AAA management to automate the segmentation of aneurysm morphology from CT and ultrasound images, allowing for more precise risk assessment and monitoring [13].

CNNs are highly effective at processing medical imaging data. Characteristics such as size, thrombus distribution, and wall stress their use in CT angiography for AAA screening has greatly enhanced early diagnosis and surveillance, assisting vascular surgeons in making clinical decisions [14].

XGBoost is known as a gradient-boosting decision tree algorithm that is particularly good at evaluating morphological, biomechanical, and organized clinical data. In AAA prediction models, it has been used to precisely stratify patients according to their rupture risk by incorporating important factors such as arterial tortuosity, intraluminal thrombus thickness, and artery wall stress [15]. A comparison of AI/ML models for AAA prediction, including their key features and accuracy, is summarized in Table 1.

**Table 1:** Comparison of AI/ML models for AAA prediction, focusing on data types, key features, accuracy, and validation ML: machine learning, APC: aneurysm prognosis classifier, DBN: deep belief network, SVM: support vector machine, AAA: abdominal aortic aneurysm References: [7-18]

Model	Data type	Key features	Accuracy (AUC/other)	Clinical validation	Reference
Gradient boosting (XGBoost)	Clinical, biological, morphometric, biomechanical	Diameter-diameter ratio, tortuosity, intraluminal thrombus thickness	81.2% (95% CI: 61.1-100%)	Tested on 40 patients, follow-up CT scans	Kontopodis et al. (2023) [15]
Support vector machines (SVM)	Clinical, biological, morphometric, biomechanical	Diameter-diameter ratio, tortuosity, intraluminal thrombus thickness	68.8% (95% CI: 46.5-91%)	Tested on 40 patients, follow-up CT scans	Kontopodis et al. (2023) [15]
Geometric and biomechanical modeling with ML	CT imaging, biomechanical parameters	Lumen diameter, peak wall stress, intraluminal thrombus	AUC 0.85 (vs. 0.80 for baseline diameter)	Tested on 189 patients, improved prediction of rupture and surgical threshold	Liljeqvist et al. (2021) [7]
Deep belief network (DBN)	Longitudinal CT imaging, in-silico data	Patient-specific AAA expansion prediction, inscribed sphere method	Relative error of 3.1%, 65% improvement over the classical model	Validated on 20 patients' CT scan follow-ups	Jiang et al. (2019) [6]
Aneurysm prognosis classifier (APC)	Clinical, morphological, biomechanical	Maximum diameter, ILT volume, wall stress	AUC varies based on feature selection, best with combined inputs	Tested on 381 patients with known outcomes (Stable, Repair, Ruptured)	Chung et al. (2024) [18]
Deep learning-based AAA segmentation	Time-resolved 3D ultrasound imaging	Automatic segmentation using nnU-Net architecture	Dice score: 0.91 improvement, sensitivity increase vs. conventional methods	Validated on 500 patients (2495 ultrasound images)	Maas et al. (2024) [13]

By incorporating these AI-driven techniques, predictive modeling for AAA has evolved beyond traditional statistical methods, leading to improved patient risk stratification, better surveillance strategies, and optimized intervention timing.

Predictive modeling for aortic aneurysm growth

AI has propelled the predictive modeling of aortic aneurysm growth to new heights, showcasing its potential across various ML algorithms and biomarkers. One noteworthy approach involves integrating biomechanics-based biomarkers within an Extra Trees algorithm, which has demonstrated the remarkable capability to forecast accelerated aneurysmal growth [3].

Leveraging critical features such as time-averaged wall shear stress (TAWSS), strain, and intraluminal thrombus (ILT) thickness, with TAWSS singled out as particularly influential, this method has achieved a remarkable predictive accuracy, boasting an impressive area under the curve (AUC) of 0.92 for the receiver operating characteristic (ROC) curve [3].

Complementing these findings, SHAP (SHapley Additive exPlanations) dependence plots have underscored the pivotal role of these biomarkers, especially TAWSS, in shaping the model's predictive outcomes [16].

Furthermore, SHAP and local interpretable model-agnostic explanations (LIME) serve as crucial model interpretability techniques, offering vascular surgeons transparent insights into AI-generated predictions [16]. These methods enhance trust and usability in clinical settings by providing explanations for how ML models identify critical factors in AAA growth and rupture risk assessment.

Comparative efficacy analyses have further emphasized the superiority of functional and local characterization of aortic tissue features within AI models for growth prediction, surpassing those reliant solely on geometrical assessments [15]. Notably, the development of ML algorithms such as gradient boosting (XGboost) and support vector machines (SVM) has yielded promising results, with XGboost emerging as particularly adept at distinguishing high AAA growth rates from low ones thanks to its ability to identify crucial factors like the diameter-diameter ratio, tortuosity from renal arteries to aortic bifurcation, and maximum thickness of the intraluminal thrombus [15].

Building upon these advancements, the integration of geometric and biomechanical modeling with ML has further refined predictions of AAA growth and rupture, particularly in small aneurysms. Recent studies have demonstrated that geometric parameters such as maximum anteroposterior diameter (APD), undulation index (UI), and radius of curvature (RC) play crucial roles in forecasting AAA progression [17]. A study analyzing CT scans found that UI and RC exhibited strong correlations with annual AAA growth rates, with UI showing a positive correlation (r = 0.38, P < 0.001) and RC demonstrating a significant inverse relationship (r = -0.53, P < 0.001). Using these parameters in ML models, such as logistic regression and multiple linear regression, researchers achieved an area under the receiver operating characteristic (AUROC) curve of 0.80 for predicting slow growth (<2.5 mm/yr) and 0.79 for predicting fast growth (>5 mm/yr). Moreover, the model accurately predicted growth rates within a 2 mm margin of error in 87% of cases. These findings underscore the importance of biomechanical indices, alongside lumen diameter, as pivotal predictors in refining AAA growth and rupture forecasting [17].

The aneurysm prognosis classifier (APC) represents a significant advancement in AAA management, leveraging ML to classify patients into stable, repair-required, or rupture-prone cases [18]. The validation of APC involved testing on a dataset of 381 patients, incorporating clinical, morphological, and biomechanical indices, with its AUC performance varying based on feature selection [18]. This classifier achieved high sensitivity and specificity, outperforming traditional diameter-based rupture risk assessment methods. Although early in clinical adoption, pilot studies suggest that integrating APC into electronic health records (EHRs) enhances decision-making for vascular surgeons [18]. Future studies should focus on multicenter trials to validate APC across diverse patient cohorts and refine its usability in routine clinical workflows.

Big data analytics has transformed AAA risk prediction by integrating patient-specific characteristics into predictive models. Hemodynamic factors, including peak wall stress (PWS), time-averaged wall shear stress (TAWSS), and intraluminal thrombus (ILT) thickness, play a crucial role in predicting aneurysm growth and rupture risk [3]. In addition, emerging studies highlight the importance of genetic markers, particularly collagen gene mutations, which influence aneurysm susceptibility and progression [4]. Clinical and demographic factors, such as age, smoking history, hypertension, and gender, significantly contribute to AAA risk stratification, affecting both growth prediction and intervention timing [15]. For instance, Liljeqvist et al. demonstrated that combining biomechanical indices (e.g., PWS) with lumen diameter improved rupture forecasting by 15% compared to diameter-based assessments [7].

AI has been widely applied to predict complications and long-term outcomes following endovascular aneurysm repair (EVAR). One of the primary applications is endoleak detection, where deep learning models trained on CT angiography data have achieved an AUC of 0.92, significantly reducing diagnostic delays and improving surveillance accuracy [19]. In addition, predictive models incorporating patient demographics, comorbidities, and procedural details have shown strong performance in forecasting long-term survival post-EVAR, allowing for personalized postoperative care planning [19]. These AI-driven advancements have empowered clinicians to tailor postoperative monitoring strategies, ensuring improved patient outcomes.

Natural language processing (NLP) has emerged as a valuable tool for analyzing large volumes of imaging reports, optimizing AAA surveillance beyond traditional manual review methods. NLP-based algorithms have demonstrated a 40% reduction in manual review time, improving workflow efficiency [20]. Furthermore, NLP helps minimize diagnostic errors, with McLenon et al. reporting a 20% reduction in missed AAA diagnoses using NLP-enhanced radiology review systems [20]. By automating the extraction of critical findings from radiology reports, NLP enhances AAA detection, monitoring, and early intervention strategies.

As AI becomes increasingly integrated into clinical decision-making, concerns regarding professional liability and regulatory oversight are becoming more prominent. Recent legal precedents emphasize the need for clear AI accountability frameworks, particularly in cases where AI misdiagnoses impact patient outcomes [21]. One notable example involved a lawsuit regarding AI-driven misdiagnosis, underscoring the necessity of shared responsibility between developers and clinicians [22]. Regulatory bodies such as the U.S. Food and Drug Administration (FDA) have introduced stringent validation and transparency requirements for AI-driven medical devices, ensuring clinical safety and ethical implementation [22]. In addition, organizations like the American Medical Association (AMA) are actively developing ethical guidelines and liability frameworks to support responsible AI integration in healthcare [23].

These advancements collectively highlight AI's transformative potential in AAA management, enabling more precise risk assessment and individualized patient care.

AI strategies for assessing aneurysm rupture risk

AI is spearheading increasingly sophisticated strategies for assessing the risk of aneurysm rupture, particularly focusing on multidimensional data analysis and continuous model refinement. Advancements in this field are paramount for predicting the risk of AAA rupture [19]. First, comprehensive patient profiles are integral, with AI models amalgamating diverse data, including demographics, lifestyle factors, detailed medical histories, and imaging data, to create a holistic view of rupture risk. Critical analysis of imaging data through AI facilitates the assessment of aneurysm characteristics such as size, shape, and growth rate, which are pivotal indicators of potential rupture. Advanced image processing techniques allow AI algorithms to extract crucial features from medical images, including measurable aspects like diameter, area, volume, and specific shape descriptors. These AI models undergo rigorous training and validation on diverse datasets, ensuring robustness and reliability [12].

AI algorithms have demonstrated high sensitivity (>90%) and specificity (>85%) in distinguishing early-stage AAAs from normal anatomical variations [13]. For example, deep learning models trained on large datasets achieve accurate segmentation of small aneurysms, with Dice scores improving to 0.91 using nnU-Net architectures [18]. However, false positives remain a challenge due to anatomical variability among patients, necessitating extensive training datasets annotated by experts [13]. Addressing these challenges is crucial for ensuring reliable early detection and minimizing unnecessary interventions. Strategies to reduce false positives include improving dataset diversity and leveraging explainability techniques like SHAP and LIME to identify biases in model predictions [16].

Performance evaluation metrics such as accuracy, precision, recall, F1-score, and area under the ROC curve are meticulously employed to ascertain the model's clinical efficacy. Risk stratification based on AI predictions aids clinicians in determining the urgency and type of intervention required, optimizing patient management and follow-up. Importantly, AI models are not static entities; they continually evolve through continuous learning, incorporating new data to refine predictions and adapt to emerging clinical insights, thus ensuring relevance and accuracy over time. Several innovative AI-powered tools and models have emerged, including Viz AAA, which automates CT angiography assessment for timely AAA detection, enhancing early intervention strategies [13].

Deep learning techniques such as CNNs are revolutionizing AAA management, offering high accuracy and reliability in medical imaging analysis from initial screening to postoperative prognosis. In addition, solutions like ARVA provide standardized, automatic measurements of the aorta with precision comparable to that of a physician's analysis, promising improved patient monitoring and outcomes through longitudinal comparisons. These advancements underscore the transformative potential of AI in enhancing the precision, efficiency, and effectiveness of AAA rupture risk assessment, ultimately improving patient care and outcomes in clinical practice [12-13].

The cost of implementing AI in AAA screening must be considered. Studies have shown that AI can reduce imaging analysis time, improving efficiency and lowering repeat scan costs. Cost-effectiveness evaluations of the UK Abdominal Aortic Aneurysm Screening Programme (NAAASP) indicate that screening remains highly cost-effective, with an incremental cost-effectiveness ratio (ICER) of £7,370 per quality-adjusted life year (QALY) gained [20]. In addition, modeling has demonstrated that modifying surveillance intervals can further improve cost-effectiveness, with potential savings of up to £580,000 per annual screening cohort while maintaining clinical safety [20].

AI and ML enhancements in 3D ultrasound imaging for aortic aneurysms

The analysis of three-dimensional (3D) ultrasound images for the diagnosis and treatment of AAAs has greatly improved with recent developments in AI and ML. Two-dimensional (2D) ultrasound has historically been used to evaluate AAAs, with an emphasis on the maximum aneurysm diameter as a predictor of rupture risk. Nevertheless, this approach ignores biomechanical stress distribution, intraluminal thrombus volume, and complicated aneurysm morphology, all of which are critical in assessing rupture risk [12]. By combining mechanical stress analysis and volumetric segmentation, AI-driven 3D ultrasound imaging offers a more thorough assessment. Different 3D ultrasound methods for AAA imaging, along with their advantages and limitations, are outlined in Table 2.

**Table 2:** Comparative analysis of 3D ultrasound methods for AAA imaging. AAA: abdominal aortic aneurysm, EVAR: endovascular aneurysm repair References: [4,31-39]

Category	Mechanical transducer	Matrix transducer	Freehand magnetic	Freehand optical
Technical Specs	Motor-driven single crystal, sequential 2D stacking	Grid of ~9,000 crystals, real-time 3D	Magnetic tracking with 2D probes	Optical tracking with 2D probes
Advantages	High resolution, consistent alignment	Dual-plane views, fast acquisition, ergonomic	Large volume capture, flexible scanning, cost-effective	High tracking accuracy, captures complex anatomy
Disadvantages	Limited coverage, slow acquisition, motion blur	High cost, slightly lower resolution, pacemaker contraindications	Magnetic interference, probe pressure artifacts	Line-of-sight issues, postprocessing complexity
Clinical use	AAA diameter measurement, small aneurysm analysis	EVAR surveillance, endoleak detection	Whole AAA imaging, intraoperative use	Imaging around calcifications, rupture risk prediction
Limitations	Not suitable for large AAAs	Limited by grid size	Requires post-processing expertise	Technically demanding for routine use

AAAs have been successfully segmented from time-resolved 3D ultrasound (3D+t US) images using deep learning-based techniques, especially the nnU-Net architecture [13]. AI-driven models have exhibited superior performance in terms of accuracy, sensitivity, and stability, while also being noninvasive and cost-effective for clinical implementation. These techniques leverage the coherence between consecutive ultrasound slices, improving volume quantification and enabling personalized mechanical modeling of aneurysms, leading to enhanced patient-specific care [11].

A study by Maas et al. investigated the application of deep learning in AAA segmentation using a dataset of 500 patients, resulting in 2,495 3D+t US images [13]. Four different deep learning models, based on the nnU-Net framework, were trained and validated against conventional CT scans and expert reader evaluations. The deep learning models significantly outperformed traditional non-deep learning segmentation methods, achieving higher Dice scores, accuracy, and sensitivity. The most effective model was the full-resolution model without data augmentation, which demonstrated superior segmentation performance and reduced the rate of poor segmentation scores compared to conventional methods.

AI tools are increasingly being integrated into radiology workflows to enhance efficiency and accuracy. Platforms like Aidoc’s BriefCase prioritize urgent cases, allowing radiologists to focus on critical findings [23]. Similarly, AI-driven platforms automate tasks such as CT angiography assessment, reducing manual workload without compromising diagnostic accuracy [23]. These tools are typically integrated into existing radiology software, requiring minimal additional infrastructure. Clinicians interact with AI-generated findings by reviewing and validating them, ensuring trust and reliability in clinical decision-making. However, challenges such as training requirements for clinicians and standardization across institutions must be addressed to facilitate widespread adoption.

These findings align with broader research on AI applications in vascular imaging, where deep learning models have shown promise in improving diagnostic accuracy and treatment planning for aortic aneurysms. AI-based predictive models have been explored for risk stratification, aneurysm growth prediction, and postoperative outcome forecasting [11-12]. The integration of deep learning in 3D+t US imaging represents a significant step toward enhancing AAA monitoring, allowing for more precise assessments of aneurysm stability and rupture risk.

By leveraging AI-enhanced 3D ultrasound imaging, clinicians can move beyond diameter-based AAA monitoring toward a more comprehensive and individualized approach, ultimately improving patient outcomes and optimizing clinical decision-making.

ML models and aneurysm outcomes

Recent developments in ML and AI have greatly enhanced the detection, tracking, and treatment of aortic aneurysms. Using CT scans to screen for AAAs has proven to be a particularly successful and feasible use of deep learning-based segmentation pipelines. For the early detection of aortic aneurysms, these AI-driven pipelines are essential because they allow for prompt intervention and enhance patient outcomes [18-22].

ML for AAA Screening and Diagnosis

To diagnose AAAs from CT scans, deep learning models, particularly CNNs, have shown exceptional sensitivity and specificity. AAAs from computed tomography angiography (CTA) can be detected with automated AI-based screening, which greatly reduces human labor while preserving good diagnostic accuracy [23]. Clinical decision-making is further aided by prediction, focusing on data types, key features, accuracy, and validation. Algorithms use deep learning to evaluate aneurysm development rates and the necessity of surgical intervention [23]. Beyond diagnosis, ML models can estimate aneurysm growth rates and predict the need for surgical intervention. Advanced deep learning algorithms, such as fully automated segmentation pipelines, have shown the ability to accurately detect and measure AAAs, offering precise quantitative assessments of aneurysm size, thrombus burden, and calcifications [24]. These developments are crucial in refining surgical decision-making and optimizing patient-specific treatment strategies.

ML in AAA Treatment Planning and Prognosis

ML has also been applied to assess treatment outcomes and determine the most effective intervention strategy. AI models have been used to compare the effectiveness of EVAR versus open surgical repair, providing data-driven insights that suggest EVAR yields better survival rates for certain patient groups [17]. In addition, predictive models leveraging big data analytics assist in formulating individualized treatment plans by simulating the outcomes of potential therapeutic interventions. One notable advancement in this field is the APC, an ML-based tool that evaluates multivariate patient data to categorize AAAs into stable, repair-required, or rupture-prone cases. This classifier serves as a valuable prognostic tool, aiding in clinical decision-making by identifying high-risk patients who may require closer monitoring or early surgical intervention [18].

AI in Aortic Aneurysm Surveillance and Long-Term Management

ML models are now being integrated into multiple aspects of AAA management, ranging from initial screening to long-term monitoring. AI-driven tools are capable of continuously analyzing patient data, predicting aneurysm progression, and assessing postoperative outcomes. NLP algorithms have also been employed to extract and analyze large volumes of imaging reports, improving AAA surveillance and patient follow-up strategies [25]. The integration of AI into aortic aneurysm management aligns with the principles of personalized medicine, as these technologies enable clinicians to tailor interventions based on individual patient profiles. As AI-driven models continue to evolve, their application in vascular surgery is expected to further enhance the precision, efficiency, and effectiveness of AAA diagnosis and treatment.

Challenges in AI-based predictive modeling

AI in vascular surgery brings a range of complex issues that are best described as a set of complex processes that require careful analysis and solving. The main challenge among these are issues to do with data privacy and security due to the accumulation of large volumes of patient data into regional and central databases. The threat of breaches, as well as unauthorized access, is always present, which leads to the search for effective security [26].

Furthermore, the integration of AI in clinical practices also poses questions regarding the costs of the new models of care and responsibility for the devices, which further emphasizes the need for proper economic evaluation and ethical consideration [27]. Ethical questions, especially those related to the right use of AI-based solutions in health decision-making processes, strengthen the call for ethical standards and enhanced supervision procedures [28].

Similarly, issues of data quality and access remain as there are major issues with regard to feeding ML algorithms with data to manage diseases that are rare and require more data for adequate management. Data standardization issues, input formats, and storage factors make it difficult for different AI systems to interface with each other thus the need to strive for standardization [29]. With the active participation of AI in clinical decision-making, the questions of professional liability arise, which leads to the reconsideration of the nature of the practitioner-patient relationship and the allocation of responsibility.

Algorithmic bias is a constant concern, which means that careful and highly conscious efforts must be made to ensure that the current model training is free from bias. Moreover, continuous monitoring of the safety/accuracy of the algorithms after the implementation of AI systems is required, due to the changes that the updates of the major systems can cause to the algorithms. Preserving the patient’s rights and their data from cyber threats is an essential reminder of why adequate protection plans are necessary. These are complex issues that require an integrated and complex solution involving technical, moral, and legal solutions to implement an AI system in the cardiology field without harming the patients’ rights and undermining ethical principles [30].

Emerging trends in AI and future directions

The field of cardiology is going through one of the most comprehensive paradigm shifts due to the development of breakthrough trends in AI that are going to revolutionize the way of new predictive modeling of patients. These trends include a wide range of technological advances and collaborative projects that are likely to revolutionize the sphere of CVD management [31].

Particular importance in this regard is the implementation of big data that combines genomics, transcriptomics, and proteomics in AI models, which has led to new insights into the development of aortic aneurysms and the creation of effective prevention and treatment programs. While wearable technology has gained traction in cardiovascular disease management, its role in AAA detection remains limited. However, emerging AI-driven remote monitoring techniques, including wireless implantable medical sensors (WIMS) and pressure-sensing devices, are being explored for post-EVAR surveillance. These devices provide continuous monitoring of aneurysm size and intra-sac pressure, potentially reducing reliance on periodic imaging modalities such as CT angiography and duplex ultrasound [32-33]. The comparison between traditional statistical methods and AI/ML approaches in AAA prediction is presented in Table 3.

**Table 3:** Traditional statistics vs. AI/ML in AAA AAA: abdominal aortic aneurysms, AI: artificial intelligence, ML: machine learning, CT/US: computed tomography/ultrasound References: [4,35-39]

Aspect	Traditional statistics	AI/ML
Primary goal	Validate diameter thresholds (e.g., ≥5.5 cm repair criteria)	Predict rupture risk and automate endoleak detection
Data used	Structured data such as diameter measurements and patient demographics	3D imaging (CT/US) for deep learning models
Strengths	Provides clear, guideline-driven criteria for intervention based on established diameter thresholds	Capable of handling complex patterns in imaging data, enabling real-time analysis and potentially improving diagnostic accuracy
Limitations	Relies on linear assumptions and may be constrained by small datasets, potentially limiting the ability to capture the complex nature of aneurysm progression and rupture risk.	"Black-box" models; require large datasets.

WIMS designed for AAA monitoring utilize inductive coupling mechanisms to track aneurysm expansion in real time. A recent study by Silva et al. (2024) introduced a novel implantable.

Z-shaped WIMS can detect size variations in the aneurysm sac, offering a promising alternative for long-term surveillance. Similarly, implantable pressure sensors, such as the EndoSure and ImPressure devices, have demonstrated the ability to measure intra-sac pressures and detect potential endoleaks following EVAR [33]. Drawing on the big data analytics capabilities of AI, healthcare staff are using the extensive databases of patients’ information, such as medical records, genomic characteristics, and monitoring data from wearable devices, to detect even the first signs of CVDs with great accuracy, which in turn allows for timely and targeted actions [18].

The integration of WIMS and pressure-sensing devices into routine clinical practice presents several challenges. Continuous calibration of these sensors is critical to ensure accurate measurements, as drift over time can lead to unreliable data [32]. For instance, implantable pressure sensors like EndoSure require precise recalibration to reliably detect intra-sac pressures post-EVAR [33]. Wireless data transmission from these devices may also face interference or signal loss, particularly in environments with electromagnetic noise. Inductive coupling mechanisms, such as those used in Z-shaped WIMS, help mitigate this issue but necessitate robust infrastructure for real-time monitoring [33]. In addition, patient compliance with follow-up protocols remains a concern, as non-adherence to monitoring schedules or improper handling of wearable components can compromise data quality [2]. Strategies to enhance patient engagement, such as user-friendly interfaces or automated reminders, could address these challenges and improve the reliability of remote monitoring systems.

Moreover, AI in the form of image analysis is transforming medical images to help in the evaluation, diagnosis of abnormalities, monitoring of disease states, and devising approaches to management. These developments are accompanied by AI-supported wearable gadgets, which improve the distant supervision of cardiovascular health indicators and early identification of anomalies. Furthermore, AI is helping the progress in drug discovery by using molecular information to estimate the possible therapeutic agents with novel approaches to CVD treatment [18]. Regarding the cardiovascular field, the role of AI is greatly expanding in the diagnosis and prognosis, which has larger application prospects for providing accurate diagnosis and prognosis evaluation for patients in a short time and improving the prognosis of patients. Introducing the AI systems as complementary to the physicians, the future of cardiovascular treatment is to combine data analysis with the physicians’ recommendations, thus paving the way to the new paradigm of highly integrated and individualized treatment. Besides vascular surgery, AI has the opportunity to revolutionize many other areas of healthcare such as medical imaging, drug discovery, telemedicine, remote monitoring, robotic surgery, individualized medicine, and analytics, which proves that it has a crucial place in the development of the modern healthcare system [12].

The integration of multi-omics data, including genomics, transcriptomics, and proteomics, into AI models, has shown promise in advancing AAA prediction and management. ML techniques such as principal component analysis (PCA) and autoencoders are pivotal in combining these datasets while reducing noise [34-39]. PCA reduces dimensionality by identifying the most significant features, while autoencoders learn compressed representations of data, minimizing noise and redundancy. Feature selection algorithms like XGBoost and random forests excel at identifying key biomarkers across omics layers, enabling the development of predictive models that account for genetic predispositions and molecular pathways involved in AAA progression [4]. These approaches allow for a more comprehensive understanding of disease mechanisms and facilitate personalized treatment strategies. For instance, multi-omics models have identified novel biomarkers associated with AAA rupture risk, such as specific gene expression patterns linked to inflammatory pathways. However, integrating multi-omics data poses challenges, including data heterogeneity and missing values, which require advanced imputation techniques and robust preprocessing pipelines.

AI has also shown significant potential in drug discovery and repurposing for cardiovascular diseases. Platforms like Atomwise leverage AI to identify novel biomarkers, accelerating therapeutic development [31]. In addition, AI facilitates the repurposing of existing drugs, such as statins, for their anti-inflammatory effects in AAA treatment [19]. These advancements underscore AI’s transformative role in personalized medicine, enabling tailored therapies based on patient-specific factors like genetics and medical history.

Thus, there is nothing but great potential for AI to transform cardiovascular practice by automating administrative work, advancing the clinical application of research findings, patient involvement, and clinician training with the aim of improving cardiovascular health and quality of life for millions of people around the globe [12].

## Conclusions

In conclusion, the detailed analysis of sophisticated ML algorithms underscores their potential to refine diagnostic accuracy, enhance treatment strategies, and establish innovative cardiac care methodologies. It articulates the significance of integrating AI with traditional clinical practices, highlighting the emergence of predictive analytics as a pivotal asset in the advancement of cardiology.

Despite these advancements, key barriers remain, including clinician trust, regulatory compliance, and real-world implementation hurdles. Concerns such as algorithmic bias, data security in AI-driven diagnostics, and liability issues in AI-assisted decision-making must be addressed to ensure the responsible deployment of these technologies. The development of ethical AI governance models and robust validation frameworks will be essential in overcoming these challenges.

The future of AI in cardiology is expected to bring significant advancements, including AI-driven clinical decision support, the integration of multimodal data for comprehensive patient assessment, and real-time AI monitoring for cardiovascular patients. Multidisciplinary research efforts, collaboration between AI developers and clinicians, and improved regulatory guidelines will play a crucial role in fostering the safe and effective adoption of AI in clinical settings.

As research continues to evolve and AI becomes further embedded in clinical practice, the importance of continuous learning and adaptation cannot be overstated. The journey toward optimizing patient outcomes in cardiology with AI is ongoing, inviting professionals within the field to contribute to this dynamic and evolving discourse.
